# Wafer-scale Fabrication of Non-Polar Mesoporous GaN Distributed Bragg Reflectors via Electrochemical Porosification

**DOI:** 10.1038/srep45344

**Published:** 2017-03-27

**Authors:** Tongtong Zhu, Yingjun Liu, Tao Ding, Wai Yuen Fu, John Jarman, Christopher Xiang Ren, R. Vasant Kumar, Rachel A. Oliver

**Affiliations:** 1 Department of Materials Science and Metallurgy, University of Cambridge, 27 Charles Babbage Road, Cambridge, CB3 0FS, United Kingdom; 2 Nanophotonics Centre, Cavendish Laboratory, University of Cambridge, CB3 0HE, United Kingdom; 3 Department of Electrical and Electronic Engineering, The University of Hong Kong, Pokfulam Road, Hong Kong

## Abstract

Distributed Bragg reflectors (DBRs) are essential components for the development of optoelectronic devices. For many device applications, it is highly desirable to achieve not only high reflectivity and low absorption, but also good conductivity to allow effective electrical injection of charges. Here, we demonstrate the wafer-scale fabrication of highly reflective and conductive non-polar gallium nitride (GaN) DBRs, consisting of perfectly lattice-matched non-polar (11–20) GaN and mesoporous GaN layers that are obtained by a facile one-step electrochemical etching method without any extra processing steps. The GaN/mesoporous GaN DBRs exhibit high peak reflectivities (>96%) across the entire visible spectrum and wide spectral stop-band widths (full-width at half-maximum >80 nm), while preserving the material quality and showing good electrical conductivity. Such mesoporous GaN DBRs thus provide a promising and scalable platform for high performance GaN-based optoelectronic, photonic, and quantum photonic devices.

Distributed Bragg reflectors (DBRs) can be considered as one-dimensional photonic crystals which consist of alternating layers with high- and low-refractive index. High quality and high reflectivity DBR-based semiconductor microcavities are essential building blocks for the development of various optoelectronic devices, such as highly efficient microcavity LEDs^1^, exciton-polariton lasers^2^, and vertical-cavity surface-emitting lasers (VCSELs)^3^.

III-nitride materials have not only achieved commercial success in the realization of highly efficient photonic devices including light emitting diodes (LEDs)^4^ and lasers^5^, but also exhibit particular advantages for the development of quantum light sources^6^, and the exploration of quantum information science^7^,^8^,^9^ and light-matter interactions^2^,^10^. However, in the context of both LEDs and quantum light sources, the conventional polar nitrides exhibit limitations due to the large polarization-induced internal electric fields arising in heterostructures which reduce the spatial overlap of the electron/hole wavefunctions. Non-polar III-nitride materials promise not only the advantage of reduced internal electric fields and thus higher rates of radiative recombination for improved device efficiencies, but also additional unique properties such as the emission of strongly linearly polarised light due to the presence of anisotropic biaxial strain in the asymmetric non-polar crystal. A high degree of polarisation has been achieved not only in the emission from non-polar quantum wells, with potentially wide-ranging application in displays^11^,^12^, but also in single non-polar quantum dots, whose polarised emission could be utilised in quantum key distribution protocols^13^. In both these applications, non-polar DBRs could be used to improve light extraction, increasing device efficiency, with the development of micro-pillar cavities providing a potential route to light-matter coupling in the quantum dot case. Non-polar VCSELs utilising DBRs, could provide additional degrees of freedom in laser design^14^, enhanced radiative efficiencies and higher optical gain^15^. However, the conventional approach to DBR fabrication in the polar nitrides, using alternating layers of different nitride alloys with different refractive index, is extremely challenging in the non-polar orientations^16^,^17^, since there is no available alloy that will lattice match to non-polar GaN^18^ (*c*-plane GaN can be lattice matched by the low-index In_0.18_Al_0.82_N^19^,^20^,^21^.). Therefore, there is no simple epitaxial strategy to achieve crack-free and high reflectance non-polar GaN-based DBRs.

As a result, the first violet non-polar VCSEL structure with inherent polarisation locking was not demonstrated until 2012 by Holder *et al*.^15^. The processing of the device involved the removal of an InGaN sacrificial layer by photoelectrochemical (PEC) etching and the deposition of top and bottom dielectric DBR mirrors. Whilst this process allows good control of the cavity length, it also involves complex and challenging fabrication. More importantly, the PEC etching is not only known to be dopant selective and bandgap selective, but also known to be extremely sensitive to structural defects^22^. Threading dislocations are known to act as charge trapping sites^23^, and prevent the diffusion of photo-generated electron-hole pair and subsequently lead to unetched material, such as the formation of whiskers^24^. This limits the application of such PEC method to the use of GaN substrates with very low defect densities, i.e. bulk or free-standing GaN substrates. Recently, Tao *et al*. have demonstrated a non-polar air-gap DBR structure by *in-situ* thermal decomposition of the GaN layers^25^ and achieved strong coupling in non-polar GaN/AlGaN microcavity with air/AlGaN DBRs at room temperature^26^. Although this air-gap approach is promising to overcome the low-refractive index contrast in conventional GaN-based DBRs, it involves rather extensive processing steps, such as epitaxy, e-beam lithography, dry etching, and *in-situ* annealing. Perhaps more fundamentally in the context of blue or green emitters, InGaN active layers with intermediate and high indium content are known to be very sensitive to thermal damage^27^ which may limit the application of this approach. (This issue has yet to be thoroughly evaluated).

An emerging alternative approach, the development of mesoporous (MP) DBRs, has attracted increasing attention in recent years since tuning of the spectral response may be achieved by varying the porosity (and thus the refractive index contrast), the layer thicknesses, and the number of repeat periods^28^,^29^,^30^,^31^. Zhang *et al*. have proposed an electrochemical (EC) method for the fabrication of porous GaN DBRs on *c*-plane GaN^32^. The EC process utilises the epitaxial growth of lattice-matched highly n-doped and undoped GaN layers, and porosification of the n-doped layers^33^. However, due to fact that the EC porosification process is very much limited in the lateral direction, the area of the DBR region has to be patterned into ~50 μm trench openings. This limits their viability for practical optoelectronic devices on a large scale. Furthermore, the top GaN surface had to be protected by a thick silicon dioxide (SiO_2_) layer, which will induce further processing steps and constrain the material design.

In this article, we demonstrate a facile wafer-scale (2-inch) fabrication route for highly reflective non-polar *a*-plane MP-GaN DBRs that utilises a one-step selective electrochemical porosification of highly silicon doped GaN layers which proceeded both laterally and vertically. There was no additional SiO_2_ deposition to protect the top GaN surface and no extra processing/masking were carried out for the subsequent electrochemical etching process, which results in a much simplified fabrication process. GaN/MP-GaN DBR mirrors with high reflectivities (>96%) and wide spectral stop-band widths are achieved. Structural and optical characterization confirm a uniform porosification process that is unaffected by the presence of structural defects and the preservation of the structural quality of the material. Hence, the etched structures are amenable to regrowth of further GaN-based materials or deposition and patterning of other materials. Due to the good vertical electrical conductivity and the ability to tune the spectral response across the visible by varying the epitaxial GaN layer thicknesses, such MP-GaN DBRs provide a new platform for a whole host of technological possibilities including GaN-based microcavity structures for electrically driven VCSELs and quantum light sources.

## EC porosification and structural characterization of GaN/MP-GaN DBRs

**Figure 1:**
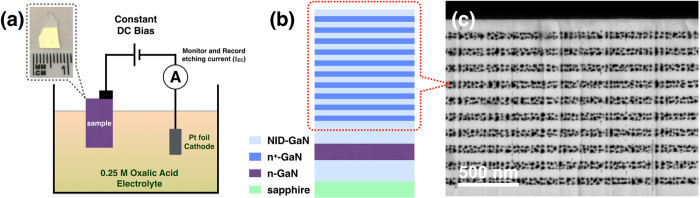
(**a**) A simple schematic of the experimental setup for the EC etching and a photograph of a sample after the etching under room light illumination. (**b**) Schematic of the DBR structure. (**c**) Cross-sectional SEM image of the 10 pair GaN/MP-GaN DBR structure.

Figure 1 shows a schematic of the EC experimental setup. The EC etching process carried out in a constant voltage mode (with a DC bias of 6 V) and controlled by monitoring and recording the etching current signal at room temperature without UV illumination. The EC porosification process begins with the oxidation of the alternating n^+^-GaN layers by localised injection of holes upon the application of a positive anodic bias, and localised dissolution of such oxide layer in the acid-based electrolyte will result in the formation of mesoporous structure^32^,^34^. The end of the anodisation process is reached when the etching current drops to the base line level, indicating that all the n^+^-GaN layers have been etched and transformed into mesoporous GaN layers, typically after 30 minutes. A photograph of a GaN/MP-GaN porous DBR sample after the EC etching is also shown in Fig. 1a, which demonstrates the high uniformity of the EC etching process across the entire sample area that has been immersed in the etching solution (~1 × 1 cm^2^).

The schematic of the epitaxial non-polar sample structure is shown in Fig. 1b, which consists of alternating non-intentionally doped GaN (NID-GaN) and n^+^-GaN layers. The cross-sectional scanning electron microscopy (SEM) image in Fig. 1c shows the morphology of the porous DBR structure, which was taken from an edge cleaved post-etching, far away from the original sample edges. This confirms that the porosification process proceeded extremely uniformly across the entire sample area being immersed in the etching solution and the etched layer morphology is indeed mesoporous (d ~30 nm). The figure shows that the NID-GaN layers stay almost intact during the EC etching. Only the n^+^-GaN layers were selectively etched and transformed into mesoporous layers.

**Figure 2:**
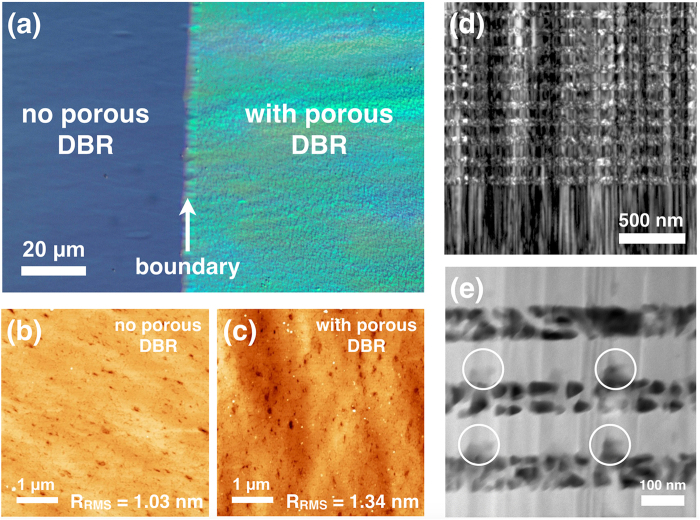
(**a**) A top-view Nomarski optical image of the GaN/MP-GaN DBR sample, where a darker non-porous region and highly reflective porous region can be seen (the boundary is marked by the white arrow). AFM images taken from regions (**b**) without porous DBR and (**c**) with porous DBR. (**d**) Weak-beam dark-field TEM image taken along [0001] using **g** = 11–20. (**e**) HAADF-STEM image of the MP-GaN DBR structure. The white circles indicate the positions where the NID-GaN layers have also been etched due to the vertical etching component of the EC porosification process.

Figure 2a shows a top view Nomarski optical image of the as-etched sample, where a boundary (marked by the white arrow) that corresponds to the position of the sample being immersed in the EC etching solution can be seen. The optical contrast between the regions with and without the porous DBR arises due to the altered reflectivity of the etched region. To evaluate possible etching damage of the top surface, atomic force microscopy (AFM) images were taken from non-porous and porous regions, which are shown in Fig. 2b,c, respectively. Apart from some dirt/small particles present in the porous region that may be related to the EC etching products, contaminants in the etching chemicals and/or sample cleaning, no changes to the surface morphology were observed and the root mean square roughness (R_RMS_) of the top GaN surface is similar in both the etched and unetched regions. Hence, we find no evidence that sub-surface EC porosification degrades the GaN top surface, implying that such a porous DBR could be used as a bottom mirror template for the regrowth of other heterostructures or, for example, deposition of high quality dielectric DBRs in order to form a planar microcavity. Therefore, the porous DBR structure is formed purely by epitaxial growth of alternating NID-GaN/n^+^-GaN and by a simple EC porosification approach without the need to protect the sample surface with SiO_2_, making the sample into complex pattern structures, or UV illumination.

We further investigated the microstructure of the MP-GaN DBR sample and the impact of the structural defects on the EC porosification process by cross-sectional transmission electron microscopy (TEM). Figure 2d shows a weak-beam dark-field TEM image, which reveals the presence of structural defects (mostly basal plane stacking faults and dislocations, see *Materials growth*) that are inherent from the GaN pseudo-substrate and their propagation through the MP-GaN DBR stack to the top GaN surface. It is clearly seen in the high-angle annular dark field scanning TEM (HAADF-STEM) image in Fig. 2e, that mesopores are formed independent of the position of the defects in the n^+^-GaN layers. However, we notice that the NID GaN layers seem to have also been slightly etched at various locations (marked by the white circles), implying that in additional to the usual lateral etching pathways^32^, the EC porosification mechanism might involve another vertical etching component, which may be defect related. We estimated the density of such vertical etching pathways to be ~2 × 10^9^ cm^−2^ based on cross-sectional STEM images over a relatively large area ~2.5 μm (distance) × 0.15 μm (thickness of the TEM specimen) (see Figure S1 in Supplementary Information), which is much lower than the density of stacking faults and dislocations present in the GaN pseudo-substrate^35^. Additionally, we note that the density of vertical pathways could well be over-estimated due to the fact that the pore morphology might have been altered during the TEM sample preparation process. Nevertheless, this suggests that the most commonly found defects do not influence the EC porosification process and are not responsible for the vertical etching routes, as also evidenced by the observation of several defects in the TEM images which appear to be having no influence on the etching. Moreover, the lack of any etching below the DBR stack suggests that the doping is required in addition to the defect pathway for etching to occur. Based on the dark field TEM image shown in Fig. 2d, we estimate a dislocation density of ~3 × 10^10^ cm^−2^. Over ~80% of the dislocations present in such pseudo-substrates have been found to be partial dislocations bounding stacking faults^35^. However, a minority of the dislocations (less than 20%) are perfect threading dislocations (**a**-type, **c**-type, and **a + c** type) with a density in the 10^9^ cm^−2^ regime, similar to the vertical pathways. We consider such dislocations to be plausible candidates for the vertical porosification routes, although the current data to not allow us to distinguish whether all perfect dislocations are likely to be involved or just a sub-set with a specific Burger’s vector. In addition, TEM analysis also reveals that the fabrication of MP-GaN DBR via electrochemical porosification has no impact on the structural quality and does not affect or generate the defects, in contrast to strained epitaxial DBRs where there can be dislocation generation and/or cracking^36^.

### Optical properties of GaN/MP-GaN DBRs

**Figure 3:**
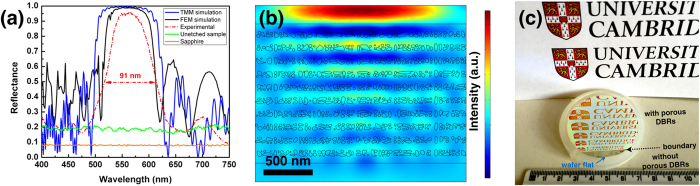
(**a**) Experimental (red dash line), transfer matrix method (blue solid line), and finite element method simulated (black solid line) reflectance spectra from the GaN/MP-GaN DBR structure. The reflectance spectra of the unetched sample (green solid line) and the sapphire substrate (orange solid line) are also shown for comparison. (**b**) Electric field distribution in a GaN/MP-GaN DBR with real pore morphology inputted by digitising the SEM image shown in Fig. 2b into the finite element model. (**c**) Photograph of an as-etched 2-inch mesoporous GaN DBR wafer reflecting a card with the Cambridge University Logo.

The reflectance spectra of the GaN/MP-GaN DBR were measured using a micro-reflectance setup using ambient room light and normalized to a commercial silver mirror with a spot size of ~1 μm. Figure 3a shows the measured reflectance spectrum of a GaN/MP-GaN DBR structure with a peak reflectance centred at ~564 nm and a stop-band with a full-width at half-maximum of 91 nm. Using the cross-sectional SEM image shown in Fig. 1c as input data for actual pore morphology and assuming the pores are axis-symmetrically distributed, a finite element simulation of the reflectance was performed and is shown as black solid line in Fig. 3a. It should be noted that the simulated peak reflectance is just over 98.7%, which is less than that of using a 1D transfer matrix method (TMM) (~99.6% - green solid line in Fig. 3a) based on a porosity of 58.74% (estimated from the SEM image) and thus an effective refractive index of 1.55 (Fig. 1c). However, in TMM the mesoporous layer is assumed to be homogeneous with a single effective refractive index, whereas the finite element method (FEM) takes into account of the spatially varying refractive index due to porosity and should better reflect the actual reflectance of the mesoporous layers. Moreover, several factors might also contribute to the marginally lower simulated peak reflectance obtained by FEM. One is the fact that the cross-sectional SEM is not perfect, and artifacts in the SEM image particularly due to the presence of cleavage steps may influence the uniformity of the observed pore morphology. Secondly, in the finite element model, the difference between the largest and smallest elements is limited, so we have had to merge some of the smaller pores for the simulation, and this will also affect the uniformity of the simulated pores. Thirdly, vertical etching pathways for the EC porosification process have been observed in Fig. 2e, the impact of such vertical etching routes (e.g. damage of the NID-GaN layers) and structural defects on the reflectivity have not been included in the FEM model. We note that further future improvements to the model could use TEM images of the pores similar to Fig. 2e, or even exploit electron beam tomography to establish the three dimensional pore structure^37^.

A peak reflectance of more than 96% is achieved on non-polar GaN/MP-GaN DBR structure with a very large spectral width, more than 80 nm. We note that the measured peak reflectance is slightly lower than that of the simulated values, which could be attributed to the local non-uniformity of the mesoporous GaN layer and the vertical etching pathways where these lead to some slight porosification of the NID GaN. Nevertheless, it is worth stressing the fact that (to our knowledge) this is the highest reported peak reflectance from a non-polar III-nitride DBR structure, and we also observe an increase of more than a factor of 2 in the stop-band width compared to previously reported structures^16^,^17^. This is attributed to the fact that a much larger refractive index contrast can be achieved using mesoporous GaN layers without introducing a significant lattice mismatch which would lead to large strains and degradation of structural quality (via the formation of cracks and generation of dislocations). In contrast, the more usual method for the fabrication of nitride DBRs, the use of Al-containing epitaxial layers on GaN, such as Al(Ga)N and InAlN to achieve a refractive index contrast, inevitably leads to significant strain in at least one in-plane direction for non-polar structures^18^,^38^. Furthermore, the finite element simulation also provides information on the propagation and scattering of an electromagnetic (EM) wave in the GaN/MP-GaN DBR structure. Figure 3b shows the distribution of electric field for a propagating EM wave, where an exponentially decaying field profile into the DBR structure can be clearly seen. Figure 3c shows the photograph of an as-etched 2-inch wafer under room light illumination and the reflection of a card printed with a logo. While the region close to the wafer-flat is transparent and unetched, the intense reflection in the DBR region demonstrates the uniform EC porosification process and the realization of high reflectance non-polar GaN/MP-GaN DBRs on a wafer-scale. Given the fact that uniform porosification occurs across the entire 2-inch wafer, it is confirmed again that the EC porosification mechanism involves both lateral and vertical etching pathways. Although the local refractive index of the NID-GaN layers might be altered by the vertical etching, we stress the fact that the measured reflectivity values are very close to the theoretical values and the density of such vertical etching pathways is considered to be low enough (~2 × 10^9^ cm^−2^), that the global reflectivity at the wafer scale (~cm) is only marginally affected and that the majority of the material should exhibit a sufficient reflectivity, unaffected by these issues, to allow the fabrication of devices such as LEDs and micropillar cavity structures for single photon sources with reasonable yield. However, we note that improved GaN pseudosubstrates with a much lower density of perfect dislocations still show porosification, even when the typical dislocation spacing is a few microns or more, which will reduce the effect of the vertical etching pathways further while still allowing wafer scale fabrication (see Figure S2 in Supplementary Information).

**Figure 4:**
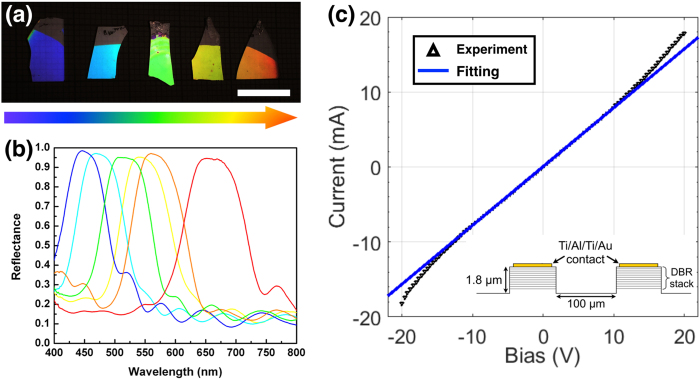
(**a**) Photographs under room light illumination (scale bar = 1 cm) and (**b**) Experimental reflectances of a number of GaN/MP-GaN DBR structures with different layer thicknesses demonstrating tunable photonic band gaps across the visible spectrum. (**c**) Current-voltage (I-V) curves of the non-polar GaN/MP-GaN DBR structure.

Figure 4a,b show the photographs under room light illumination and the measured reflectance spectra of various GaN/MP-GaN DBR structures. A widely tunable stop-band with high reflectance (>96%) across the entire visible spectrum is demonstrated, simply by varying the epitaxial layer thicknesses of the NID-GaN and n^+^-GaN. Due to the large refractive index contrast between the GaN and MP-GaN layers, the stop-band widths are also maintained to be very wide (>80 nm).

### Electrical properties of GaN/MP-GaN DBRs

To evaluate the electrical properties and vertical conductivity of the MP-GaN DBR structure, 400 × 400 μm mesas were etched to a depth of 1.8 μm, and Ti/Al/Ti/Au contact pads were deposited on the top surface. The current-voltage (I-V) behaviour was then measured between two mesas separated by 100 μm, as shown in Fig. 4c. The fit to the central region gives a series resistance of 1.2 kΩ. Transmission-line measurements were also performed, by measuring the resistance between mesas separated by different distances. Extrapolating to zero separation gives a contact resistance of 1.1 ± 0.2 kΩ, suggesting that the measured resistance is dominated by the MP-GaN DBR and the ohmic contacts, and not by the sheet resistance of the material between mesas. Nevertheless, these measurements show that the non-polar GaN/MP-GaN DBR is indeed electrically conductive, which will be compatible with electrically driven GaN optoelectronic devices.

## Conclusions

In conclusion, we present a simple one-step electrochemical process for wafer-scale fabrication of highly reflective and conductive non-polar GaN/mesoporous GaN DBRs via selectively porosifying the alternating Si-doped GaN layers both laterally and vertically. While remaining perfectly lattice-matched, stress free, and preserving the material quality, these GaN/MP-GaN DBR structures exhibit high peak reflectivity (>96%) across the visible spectrum with very wide and flat stop-bands (width >80 nm) by varying the GaN layer thicknesses. Such vertically conductive and widely tunable mesoporous GaN DBRs provide a new and viable route for realizing microcavity structures and achieving high performance GaN-based and hybrid optoelectronic devices in the near future, such as electrically injected vertical-cavity surface-emitting lasers, polariton lasers, and single photon sources.

## Methods

### Material Growth and Fabrication

The non-polar (11–20) DBR sample was grown by metal-organic vapour phase epitaxy in a 6 × 2 in. Thomas Swan close-coupled showerhead reactor on *r*-plane sapphire substrates using trimethylgallium and ammonia as precursors, hydrogen as a carrier gas and silane for n-type doping. Firstly, a 4 μm thick *a*-plane GaN pseudosubstrate was grown with a nominal dislocation density of ~4 × 10^9^ cm^−2^, and a basal plane stacking fault density of ~5 × 10^5^ cm^−1^, in which a single SiN_x_ interlayer was used for defect reduction^35^,^39^. After the growth of another 500 nm undoped GaN, 10 pairs of alternating n^+^-GaN and undoped GaN layers were grown to act as the DBR structure. The undoped GaN layers are non-intentionally doped with an electron concentration < 10^17^ cm^−3 ^40. The n^+^-GaN layers have a nominal silicon doping concentration of ~2.3 × 10^19^ cm^−3^.

### Electrochemical porosification

EC etching experiments were conducted in a two-electrode cell at room temperature with n-doped GaN as the anode and a platinum foil as the counter electrode (cathode). Oxalic acid (0.25 M) was used as the electrolyte. The anodisation process was carried out in a constant voltage mode controlled by a Keithley 2400 source meter. After anodisation, samples were rinsed with deionized water and blow dried in N_2_.

### Material Characterization

The morphology of the porous DBR structure was analysed by scanning electron microscope (Philips XL30 FEG). Scanning-TEM and dark-field TEM were performed using an FEI Company Tecnai Osiris with an extreme-FEG (X-FEG) at 200 kV. The surface morphology was studied by atomic force microscopy using a Veeco Dimension 3100 in TappingMode™. Micro-reflectance measurements were performed using a bright-field microscope (BX51, Olympus) connected with a fibre-coupled spectrometer (QE65000, Ocean Optics). A commercial silver mirror (PF10-03-P01, Thorlabs) was used for normalization.

### Simulation

A finite element simulation of the MP-GaN DBR is done using the COMSOL software. Pores are first identified from the dark areas of the SEM image (Fig. 1c), and then quadratic Bézier curves are fitted to each group of dark pixels, i.e., the pores, to trace the boundaries. The fitting results are then inputted into COMSOL, with the smallest pores being removed or merged according to the element size ratio restriction. The refractive index of GaN is inputted as a wavelength dependent Sellmeier equation according to ref. 41 while that of the pores is treated as air. Periodic boundary conditions are implemented to both sides of the GaN in the in-plane directions. And thus the reflectance of the DBR can be deduced from the scattering parameters obtained from the FEM simulations.

### Data availability

Datasets for the figures in this paper can be found at https://doi.org/10.17863/CAM.8096.

## Additional Information

**How to cite this article:** Zhu, T. *et al*. Wafer-scale Fabrication of Non-polar Mesoporous GaN Distributed Bragg Reflectors via Electrochemical Porosification. *Sci. Rep.* **7**, 45344; doi: 10.1038/srep45344 (2017).

**Publisher's note:** Springer Nature remains neutral with regard to jurisdictional claims in published maps and institutional affiliations.

## Supplementary Material

Supporting Information
