# Supplementary material for: Wafer-scale Fabrication of Non-Polar Mesoporous GaN Distributed Bragg Reflectors via Electrochemical Porosification
Source: Sci Rep. 2017 Mar 27;7:45344. doi: 10.1038/srep45344 (PMC5366952; doi:10.1038/srep45344)
Supplement: Supporting Information [file srep45344-s1.doc]

**Supporting Information**

Wafer-scale Fabrication of Non-polar Mesoporous GaN Distributed Bragg Reflectors via Electrochemical Porosification

Tongtong Zhu^1,*^, Yingjun Liu^1,*^, Tao Ding^2^, Wai Yuen Fu^3^, John Jarman^1^, Christopher Xiang Ren^1^, R. Vasant Kumar^1^, and Rachel A. Oliver^1^

^1^Department of Materials Science and Metallurgy, University of Cambridge, 27 Charles Babbage Road, Cambridge, CB3 0FS, United Kingdom

^2^Nanophotonics Centre, Cavendish Laboratory, University of Cambridge, CB3 0HE, United Kingdom

^3^Department of Electrical and Electronic Engineering, The University of Hong Kong, Pokfulam Road, Hong Kong


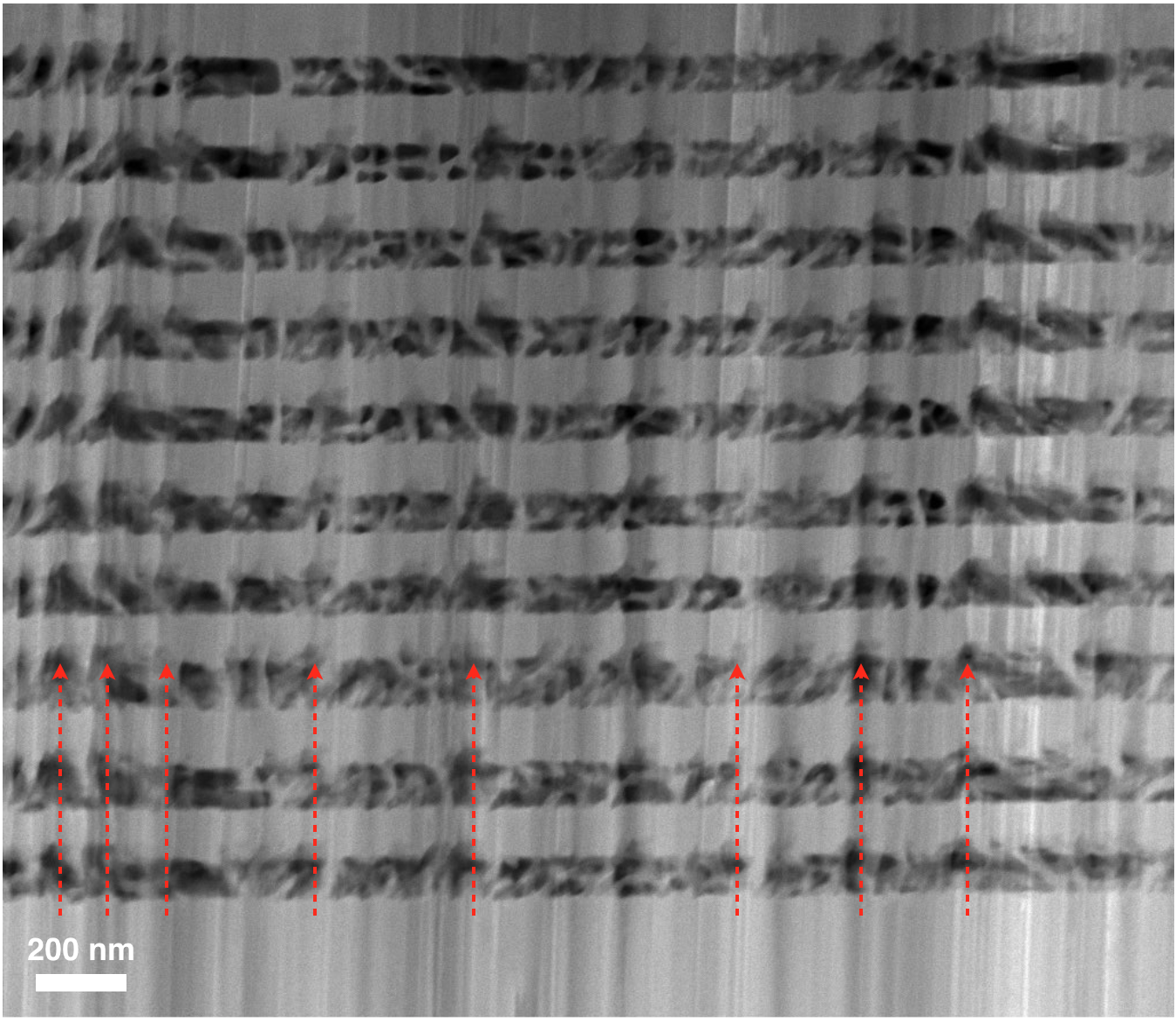


**Figure S1.** Cross-sectional STEM image of the 10 pair GaN/MP-GaN DBR structure reveals the presence of vertical etching pathways (indicated by the red dashed arrows), which shows that the NID GaN layers at these positions have also been etched. Given the length of the STEM image is 2.5 µm and the thickness of the TEM specimen is ~150 nm, we estimated the density of such vertical etching pathways at ~2 x 10^9^ cm^-2^.


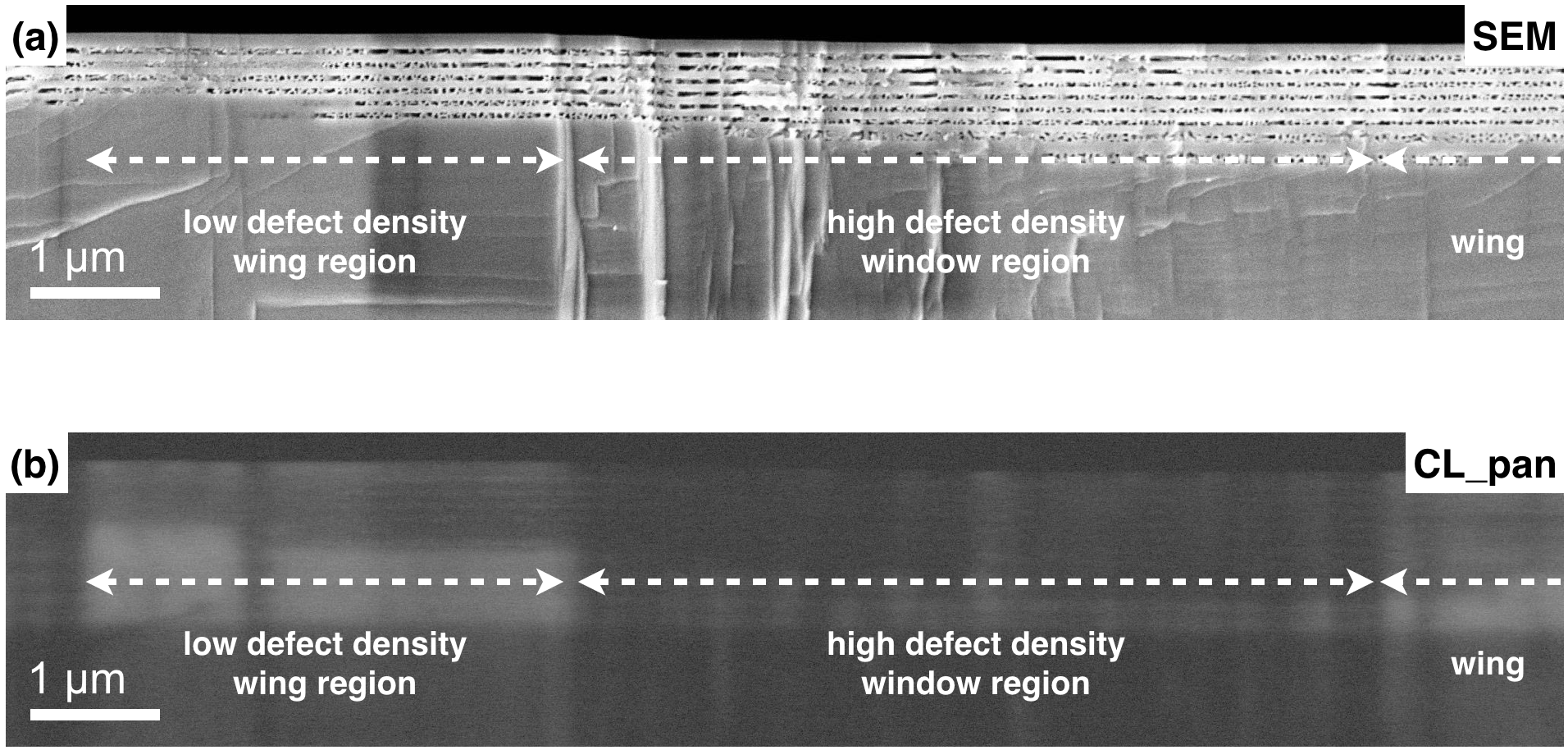


**Figure S2.** Cross-sectional (a) SEM and (b) Cathodoluminescence images of the non-polar *a*-plane GaN/MP-GaN DBR structure grown on GaN pseudosubstrates prepared via the epitaxial lateral overgrowth method.^[1]^ In the highly defective window region, the dislocation density was 1 x 10^10^ cm^-2^, and the density of basal plane stacking faults (BSFs) was 2.6 x 10^5^ cm^-2^. The low defect density wing region has a very low dislocation density <1 x 10^6^ cm^-2^, and a reduced BSF density of 2 x 10^4^ cm^-1^.^[2]^ The high defect density window and low defect density wing regions are shown to have been similarly porosified, which suggests that improved GaN pseudosubstrates with a lower density of perfect dislocations will reduce the effect of the vertical etching pathways while still allowing wafer-scale fabrication of GaN/MP-GaN DBRs.

References:

[1] Häberlen, M.; Badcock, T. J.; Moram, M. A.; Hollander, J. L.; Kappers, M. J.; Dawson, P.; Humphreys, C. J.; Oliver, R. A.; J. Appl. Phys. **2010**, 108, 033523.

[2] Emery, R. M.; Zhu, T.; Oehler, F.; Reid, B.; Taylor, R. A.; Kappers, M. J.; Oliver, R. A. Non-polar (11-20) InGaN quantum dots with short exciton lifetimes grown by metal-organic vapour phase epitaxy. *Phys. Status Solidi C* **2014**, 11, 698.
